# Fibromyalgia, Eating Disorders and Rehabilitation: The Nrf2 Link

**DOI:** 10.3390/antiox15030364

**Published:** 2026-03-12

**Authors:** Roberto Casale, Paolo Capodaglio, Kestutis Petrikonis, Antonella Paladini, Piercarlo Sarzi-Puttini, Jurga Bernatoniene

**Affiliations:** 1 Opusmedica Persons, Care & Research-NPO, 29121 Piacenza, Italy; robertocasale@opusmedica.org; 2 Research Laboratory in Biomechanics, Rehabilitation and Ergonomics, IRCCS, Istituto Auxologico Italiano, San Giuseppe Hospital, 28824 Piancavallo, Italy; paolo.capodaglio@unimi.it; 3 Department of Biomedical, Surgical and Dental Sciences, University of Milan, 20122 Milan, Italy; 4 Department of Neurology, Lithuanian University of Health Sciences, Eivenių str. 2, LT-50009 Kaunas, Lithuania; kestutis.petrikonis@lsmu.lt; 5 Department of MESVA, University of L’Aquila, 67100 L’Aquila, Italy; antonella.paladini@univaq.it; 6 Department of Rheumatology, IRCCS Galeazzi-Sant’Ambrogio Hospital, 20157 Milan, Italy; piercarlo.sarziputtini@grupposandonato.it; 7 Department of Biomedical and Clinical Sciences, University of Milan, 20122 Milan, Italy; 8 Department of Drug Technology and Social Pharmacy, Faculty of Pharmacy, Medical Academy, Lithuanian University of Health Sciences, Sukileliu pr. 13, LT-50161 Kaunas, Lithuania; 9 Institute of Pharmaceutical Technologies, Faculty of Pharmacy, Medical Academy, Lithuanian University of Health Sciences, Sukileliu pr. 13, LT-50161 Kaunas, Lithuania

**Keywords:** fibromyalgia, eating disorders, oxidative stress, Nrf2, rehabilitation, Mediterranean diet, exercise, phytochemicals

## Abstract

Background: Fibromyalgia (FM) and eating disorders (ED) represent distinct clinical entities traditionally managed within separate medical specialties, yet emerging evidence suggests significant comorbidity and potential shared pathophysiological mechanisms. Both conditions disproportionately affect women, involve complex multifactorial etiologies and substantially impair quality of life. Despite documented clinical overlaps, the mechanistic connections linking these conditions remain poorly characterized, and integrated treatment approaches are lacking. Objective: This narrative review examines the role of oxidative stress and nuclear factor erythroid 2-related factor 2 (Nrf2) pathway dysfunction as a unifying molecular mechanism connecting fibromyalgia and eating disorders, with emphasis on implications for integrated rehabilitation strategies. Methods: We synthesized current evidence on oxidative stress pathophysiology in fibromyalgia and eating disorders, focusing on Nrf2-Keap1 pathway function, clinical comorbidity patterns and rehabilitation interventions targeting antioxidant defense mechanisms. In PubMed, representative search strings included “(fibromyalgia [MeSH] OR fibromyalgia [Title/Abstract]) AND (“eating disorders” [MeSH] OR “anorexia nervosa” [MeSH] OR “bulimia nervosa” [MeSH])” and “fibromyalgia AND (“oxidative stress” OR Nrf2 OR “redox”)”. Articles in English published through December 2025 were considered, with additional records identified by manually screening reference lists. Results: Fibromyalgia patients exhibit elevated oxidative stress markers, impaired antioxidant enzyme function and compromised Nrf2 activity correlating with disease severity, with studies reporting approximately 30–50% reductions in coenzyme Q10 levels compared with healthy controls. Similarly, eating disorders demonstrate mitochondrial dysfunction and oxidative stress dysregulation, though patterns differ across eating disorder phenotypes. Nrf2 serves as the master regulator of cellular antioxidant defense, coordinating expression of over 500 genes involved in detoxification, cytoprotection, inflammation modulation and metabolic regulation. Evidence suggests Nrf2 activity is regulated by energy balance, potentially linking nutritional status with cellular stress responses. Rehabilitation interventions, including graduated exercise and nutritional optimization with Nrf2-activating foods (cruciferous vegetables, polyphenols, omega-3 fatty acids), offer mechanism-based therapeutic approaches through hormetic Nrf2 activation and direct Keap1 modification. Conclusions: Multidisciplinary rehabilitation programs integrating physical therapy, exercise prescription and nutritional strategies targeting Nrf2 activation offer evidence-based, mechanism-driven approaches to address shared oxidative stress pathophysiology. Nrf2 pathway dysfunction represents a promising and biologically plausible molecular target that may help to unify our understanding of fibromyalgia and eating disorders pending confirmation from prospective clinical studies in comorbid populations. Future research should prioritize prospective clinical trials testing Nrf2-targeted interventions in comorbid populations and collaborative patient-centered care models.

## 1. Introduction

The coexistence of fibromyalgia and eating disorders represents a clinical enigma that has received insufficient attention despite mounting evidence of their frequent comorbidity and shared pathophysiological features [1,2]. Fibromyalgia affects 2–8% of the global population, predominantly women, causing chronic widespread pain, profound fatigue and significant disability [3,4]. Eating disorders, affecting approximately 9% of individuals across the lifespan, similarly demonstrate female predominance and carry substantial morbidity and mortality [5]. While these conditions have traditionally occupied different clinical domains—rheumatology, pain medicine and psychiatry—their intersection demands prioritization in terms of gender prevalence and shared pathophysiological mechanisms [6,7].

Clinical observations reveal that individuals with fibromyalgia exhibit low resilience to stressors with elevated rates of disordered eating behaviors, including emotional eating, food avoidance and body image preoccupation [8,9,10], while those with eating disorders demonstrate higher prevalence of chronic pain conditions. The comorbidity between fibromyalgia and obesity, estimated at 40–70%, further complicates this relationship, suggesting complex bidirectional interactions between pain, metabolism and eating pathology [11,12]. Despite this clinical overlap, the mechanistic connections linking these conditions remain poorly understood, and treatment approaches continue to operate in isolated specialty silos.

This narrative review addresses a critical gap in the literature by proposing oxidative stress and Nrf2 pathway dysfunction as a unifying molecular mechanism connecting fibromyalgia and eating disorders. Nrf2, the master regulator of cellular antioxidant defense, coordinates the expression of over 500 genes involved in redox homeostasis, inflammation modulation and metabolic regulation [13,14]. Emerging evidence demonstrates impaired Nrf2 activity in fibromyalgia [15,16] and oxidative stress dysregulation in eating disorders, suggesting that these seemingly disparate conditions may reflect different manifestations of shared cellular stress response dysfunction [17].

Understanding this connection carries profound clinical implications. Recognition of Nrf2 as a molecular nexus offers a mechanistic framework for developing integrated, targeted interventions that address both conditions simultaneously through multidisciplina-ry rehabilitation strategies incorporating exercise, nutritional optimization and dietary Nrf2 activators [18,19].

## 2. Fibromyalgia and Eating Disorders: Overlapping Clinical Features

### 2.1. Fibromyalgia: Pathophysiology and Comorbidity

Fibromyalgia is a complex chronic pain syndrome characterized by widespread musculoskeletal pain, fatigue, sleep disturbances and cognitive dysfunction, affecting approximately 2–8% of the general population worldwide [3,4,20]. The condition predominantly affects women, with a female-to-male ratio of approximately 9:1 and typically manifests during middle age [4,21]. Despite extensive research, the etiology and pathophysiology of fibromyalgia remain incompletely understood, with multiple interacting factors including central sensitization, neuroendocrine dysregulation, autonomic nervous system dysfunction and systemic inflammation contributing to the clinical picture [2,15,22].

Central sensitization, a hallmark feature of fibromyalgia, involves amplification of pain and sensory signals within the central nervous system, resulting in widespread hyperalgesia and allodynia in the absence of consistent peripheral tissue pathology [2,22,23]. Functional neuroimaging studies demonstrate altered pain processing in multiple brain regions, including the anterior cingulate cortex, insula and somatosensory cortex. Neurotransmitter imbalances further compound pain dysregulation, with reduced serotonin and norepinephrine levels impairing descending pain modulation pathways, while elevated substance P levels enhance pain signal transmission.

Hypothalamic–pituitary–adrenal (HPA) axis dysregulation manifests prominently in FM patients, with many exhibiting hypocortisolism and blunted cortisol responses to stress. This neuroendocrine dysfunction contributes to fatigue, pain amplification and reduced stress resilience. Additionally, autonomic nervous system imbalance, characterized by sympathetic hyperactivity and parasympathetic hypoactivity, correlates with sleep disturbances and orthostatic intolerance.

The comorbidity profile of fibromyalgia is extensive and clinically relevant. Depression, anxiety disorders, post-traumatic stress disorder and other mood disturbances affect up to 60–80% of fibromyalgia patients [2,10]. Sleep disturbances, including non-restorative sleep and frequent awakenings, are nearly universal and contribute substantially to fatigue and pain amplification. Metabolic syndrome, obesity and insulin resistance are significantly overrepresented in fibromyalgia populations, creating a complex interplay between pain, metabolism and systemic inflammation [11,12,24,25].

### 2.2. Eating Disorders and Disordered Eating Patterns

Eating disorders, including anorexia nervosa, bulimia nervosa, binge eating disorder and other specified feeding or eating disorders [26,27], represent a distinct group of psychiatric conditions characterized by persistent disturbances in eating behaviors and associated cognitions about food, body weight and shape [5,28]. These disorders affect approximately 9% of the global population across the lifespan and are associated with significant medical complications, psychological distress and impaired quality of life. Like fibromyalgia, eating disorders disproportionately affect females, with peak incidence occurring during adolescence and early adulthood.

While fibromyalgia and eating disorders have traditionally been studied as separate clinical entities within distinct medical specialties, emerging evidence suggests significant overlap and potential shared pathophysiological mechanisms. Several studies have documented elevated rates of disordered eating behaviors in fibromyalgia populations, including emotional eating, food avoidance and preoccupation with weight and body shape [8,9,29]. Conversely, research has identified higher prevalence of chronic pain conditions, including fibromyalgia, among individuals with eating disorder.

Several factors, in various combinations and varying severity, can be connected to the association of FM and obesity, such as side effects of pharmacological treatment, thyroid dysfunction and inflammatory mediators, chronic stress and psychiatric and psychological comorbidities [6,11]. Among these, movement fear-avoidance culminating in reduction in locomotor activity, frustration over functional limitation, biomechanical/structural changes and lifestyle issues related to diet and sedentary behavior are of the utmost relevance [30,31]. Unrecognized eating disorders or disordered eating behaviors in fibromyalgia patients may complicate treatment, contribute to poor outcomes and exacerbate symptoms such as fatigue, pain and disability [8,9]. Similarly, unaddressed chronic pain in eating disorder populations may interfere with nutritional rehabilitation and psychological treatment.

### 2.3. Disturbed Eating Behaviors Beyond Classical ED Diagnoses

In recent years, increasing attention has been paid to disturbed eating behaviors that do not always meet full DSM-5 criteria but are highly relevant in chronic pain populations, including fibromyalgia [29,32]. These patterns include emotional eating, night eating, compulsive snacking and rigid “clean eating” or orthorexia-like behaviors, as well as avoidant/restrictive food intake disorder, in which selective eating, sensory hypersensitivity to textures and fear of gastrointestinal symptoms are prominent. Such subthreshold conditions can significantly affect energy balance, micronutrient status and treatment adherence, yet often remain undetected in routine rheumatology or pain clinic assessments.

Emerging data indicate that eating disorders and disturbed eating behaviors are closely linked to alterations in reward processing, interoceptive awareness and stress responsivity. Functional neuroimaging and neuroendocrine studies in anorexia nervosa and bulimia nervosa have documented abnormal mesolimbic dopamine responses to food cues, altered leptin and ghrelin signaling and changes in brain-derived neurotrophic factor, which together contribute to pathological patterns of restriction, binge eating and purging [8,33]. These mechanisms are conceptually aligned with observations in fibromyalgia, where heightened pain sensitivity, emotional distress and reduced resilience may promote emotional eating and reliance on highly palatable, energy-dense foods as a maladaptive coping strategy [8,9].

## 3. Oxidative Stress and Nrf2 Dysfunction: Unifying Mechanism

**Figure 1:**
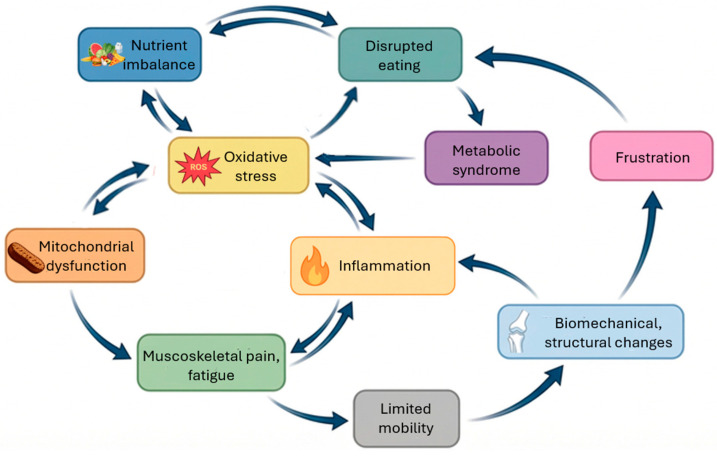
Oxidative stress is a key link in the complex regulation of fibromyalgia and eating disorders.

One of the most promising areas for understanding the connection between fibromyalgia and eating disorders lies in the role of oxidative stress and cellular redox imbalance. Oxidative stress occurs when there is an imbalance between the production of reactive oxygen species (ROS) and reactive nitrogen species and the body’s antioxidant defense mechanisms, leading to cellular damage and dysfunction. This state of redox imbalance has been implicated in the pathogenesis of numerous chronic diseases, including both fibromyalgia and eating disorders (Figure 1) [15,17,24].

While this review focuses on oxidative stress and Nrf2 pathway dysfunction as a unifying mechanistic thread, it is important to acknowledge that fibromyalgia and eating disorders share a broader landscape of overlapping pathophysiological mechanisms that together shape their clinical presentations and reinforce each other. Hypothalamic–pituitary–adrenal (HPA) axis dysregulation is well documented in both conditions, albeit with distinct directional profiles, fibromyalgia is characterized by relative hypocortisolism and blunted adrenal responses to stress, likely reflecting long-term consequences of sustained allostatic load [24], whereas eating disorders, particularly anorexia nervosa, are associated with corticotropin-releasing hormone-mediated hypercortisolism driven by severe caloric restriction, which suppresses appetite perception, promotes catabolism and disrupts energy homeostasis [27,34]. Neuroinflammation represents another shared feature in fibromyalgia. Elevated pro-inflammatory cytokines, including IL-6, IL-8, and TNF-α, have been documented alongside evidence of central neuroimmune activation [22,35] while in eating disorders, prolonged undernutrition drives altered neuroimmune signaling and disruption of the blood–brain barrier [33]. Emerging evidence further implicates gut–brain axis disruption in both conditions, with intestinal dysbiosis and impaired enteroendocrine signalling contributing to central pain sensitization in fibromyalgia [36], and to persistence of disordered appetite regulation and anxiety-related behaviors in anorexia nervosa [37]. These mechanisms do not operate independently, HPA dysregulation amplifies neuroinflammatory signalling, gut dysbiosis sustains systemic oxidative burden, and neuroinflammation feeds back onto HPA reactivity, all converging upon and modulated by redox homeostasis, positioning Nrf2 as a key regulatory node within this broader pathophysiological network rather than an isolated explanatory factor [13,14,16].

### 3.1. Oxidative Stress in Fibromyalgia

In fibromyalgia, accumulating evidence demonstrates elevated markers of oxidative stress, including increased lipid peroxidation products such as malondialdehyde and 4-hydroxynonenal, alongside reduced antioxidant capacity [15,38,39]. Recent reviews have documented that fibromyalgia patients exhibit impaired antioxidant defenses, including decreased levels of coenzyme Q10, superoxide dismutase, catalase, and glutathione peroxidase [15,40,41]. These deficiencies in antioxidant enzymes correlate inversely with disease severity measures such as the Fibromyalgia Impact Questionnaire, pain scores and anxiety levels [38,41].

Moreover, mitochondrial dysfunction has been identified as a key feature of fibromyalgia pathophysiology, potentially contributing to both oxidative stress and the characteristic fatigue experienced by patients [40,42]. Contemporary systematic reviews have systematized these findings, showing that fibromyalgia involves elevated total oxidative stress, reduced total antioxidant status and increased oxidative stress index that correlate with pain intensity and disability [15,16]. The relationship between oxidative stress and clinical manifestations in fibromyalgia appears to be bidirectional: chronic pain and stress may promote oxidative damage, while oxidative stress and mitochondrial dysfunction may exacerbate pain sensitivity, fatigue, and cognitive impairment through effects on neuroinflammation, energy metabolism and cellular signaling pathways [15,16,22].

### 3.2. Oxidative Stress in Eating Disorders

Similarly, oxidative stress has been documented in eating disorders, particularly anorexia nervosa. Studies have shown that individuals with anorexia nervosa exhibit altered mitochondrial function and increased oxidative stress in leukocytes, with evidence of mitochondrial complex I inhibition. The severe nutritional restriction characteristic of anorexia nervosa may paradoxically exacerbate oxidative stress through multiple mechanisms, including impaired antioxidant synthesis, altered energy metabolism and compromised cellular repair processes [17,43]. Interestingly, the relationship between oxidative stress and fatigue has been documented in both anorexia nervosa and fibromyalgia, suggesting a potential shared pathophysiological pathway [15,44].

More recently, oxidative stress in eating disorders has been linked to autoimmune and neuroendocrine alterations. In anorexia nervosa, elevated indices of oxidative stress and reduced total antioxidant capacity have been described alongside immune alterations, which appear to change with clinical improvement and nutritional restoration. These findings support the concept that severe and prolonged undernutrition not only disrupts mitochondrial function and redox homeostasis but may also trigger maladaptive responses affecting key brain regions involved in appetite regulation, stress responses, and energy homeostasis, further reinforcing the vicious cycle between nutritional status, oxidative damage and disturbed eating behaviors [33,43].

### 3.3. The Nrf2-Keap1 Pathway: Molecular Architecture

**Figure 2:**
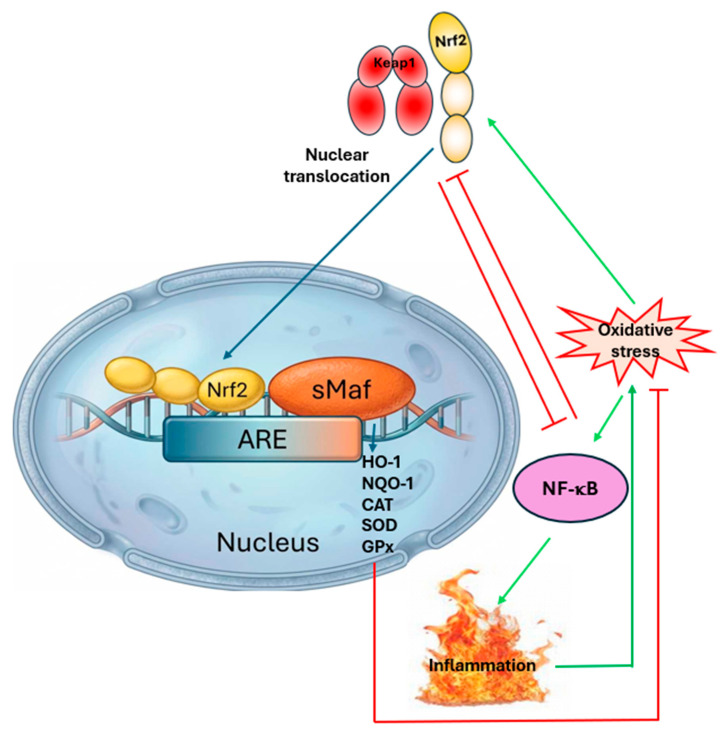
Nrf2-mediated antioxidant response and inflammatory crosstalk. Keap1—Kelch-like ECH-associated protein 1, ARE—antioxidant response element, sMaf—small Maf proteins, HO-1—heme-oxygenase-1, NQO-1—NAD(P)H quinone oxidoreductase 1, CAT—catalase, SOD—superoxide dismutase, GPx—glutathione peroxidase, NF-κB—nuclear factor kappa-light-chain-enhancer of activated B cells. Blue arrow means translocation, green arrow means activation, red blunt arrow means inhibition.

At the molecular level, the nuclear factor erythroid 2-related factor 2 (Nrf2) has emerged as the master regulator of cellular antioxidant defense and a critical mediator of the oxidative stress response [13,14]. Nrf2 is a transcription factor that, when activated, binds to antioxidant response elements (ARE) in the promoter regions of over 500 genes, thereby coordinating the expression of antioxidant enzymes, phase II detoxification enzymes and cytoprotective proteins (Figure 2) [13,14,45].

Under basal conditions, Nrf2 is sequestered in the cytoplasm by its negative regulator, Kelch-like ECH-associated protein 1 (Keap1), which facilitates its ubiquitination and proteasomal degradation, maintaining Nrf2 at low levels with a half-life of approximately 15–20 min. Upon exposure to oxidative stress or electrophilic stimuli, critical cysteine residues in Keap1 undergo modification, leading to conformational changes that disrupt the Keap1-Nrf2 interaction [46,47]. This allows Nrf2 to escape degradation, accumulate in the cytoplasm, translocate to the nucleus and heterodimerize with small Maf proteins to activate ARE-dependent gene transcription (Figure 2) [46,47].

The genes regulated by Nrf2 include those encoding superoxide dismutase (SOD), catalase (CAT), glutathione peroxidase (GPx), glutathione S-transferases (GSTs), NAD(P)H: quinone oxidoreductase 1 (NQO1), heme oxygenase-1 (HO-1) and glutamate-cysteine ligase, among many others [13,14,45]. Through this coordinated response, Nrf2 activation enhances cellular capacity to neutralize ROS, detoxify harmful compounds and restore redox homeostasis (Figure 2) [13,14].

Beyond its classical role in antioxidant defense, Nrf2 has been shown to regulate cellular processes that are highly relevant to both fibromyalgia and eating disorders, including inflammation, mitochondrial function, autophagy and metabolic regulation [13,14,48]. The Nrf2 pathway exhibits significant crosstalk with nuclear factor-kappa B (NF-κB), with Nrf2 activation generally counteracting NF-κB-driven inflammatory responses. This anti-inflammatory effect of Nrf2 is particularly relevant given that both fibromyalgia and eating disorders have been associated with altered inflammatory profiles and immune dysfunction [15,33,43].

### 3.4. Nrf2 Dysfunction in Fibromyalgia

Emerging evidence suggests that Nrf2 pathway dysfunction may play a central role in fibromyalgia pathophysiology. Recent reviews have reported that Nrf2 activity appears impaired in fibromyalgia, contributing to oxidative damage and neuroinflammation [15,16]. This impairment in Nrf2 function may explain the observed deficits in antioxidant enzyme expression and the accumulation of oxidative stress markers in fibromyalgia patients [15,38,41].

The relationship between Nrf2 and pain modulation is particularly intriguing as the transcription factor has been shown to influence nociceptive signaling through multiple mechanisms, including regulation of inflammatory mediators, modulation of mitochondrial function in dorsal root ganglia, and effects on central sensitization processes [16,22,49]. Preclinical models suggest that Nrf2 activation may have therapeutic potential in pain conditions sharing pathophysiological features with fibromyalgia [49,50]. For instance, studies in migraine models-a condition frequently comorbid with fibromyalgia—have demonstrated that Nrf2 pathway activation reduces oxidative stress and neuroinflammation in the trigeminal system, alleviating pain behaviors.

Additionally, Nrf2 activation has been proposed to reduce central sensitization, a core feature of fibromyalgia characterized by amplification of pain signals in the central nervous system [14,22]. The potential for Nrf2 activators to address both oxidative stress and pain sensitization makes this pathway an attractive therapeutic target for fibromyalgia [16,18,22,49]. Furthermore, Nrf2 appears to play a role in modulating fatigue, one of the most disabling symptoms of fibromyalgia [15,51]. The relationship between Nrf2, mitochondrial function and cellular energy metabolism suggests that impaired Nrf2 activity may contribute to the profound fatigue experienced by fibromyalgia patients [15,40,42]. Interventions that enhance Nrf2 activity, such as coenzyme Q10 supplementation, have shown promise in improving both pain and fatigue in fibromyalgia, although robust randomized controlled trials are still needed [40,41].

### 3.5. Nrf2 and Energy Balance in Eating Disorders

The role of Nrf2 in eating disorders and disordered eating behaviors is less well characterized but may be critically important. Recent research has revealed that Nrf2 activity is tightly regulated by energy-based stimuli and plays a key role in cellular responses to nutritional stress [48,52]. Dietary energy restriction, including moderate caloric restriction and certain intermittent fasting regimens, has been shown in experimental models to activate Nrf2 signaling and induce protective antioxidant responses, improving mitochondrial efficiency and reducing oxidative damage. However, these hormetic benefits appear to depend on the degree and duration of energy deficit.

The relationship between energy balance and Nrf2 varies markedly across eating disorder subtypes, reflecting distinct pathophysiological mechanisms. In anorexia nervosa (restrictive subtype), severe and sustained undernutrition depletes antioxidant substrates (including glutathione precursors, vitamins C and E, and selenium) and micronutrient cofactors essential for antioxidant enzyme function, paradoxically dysregulating Nrf2 despite theoretical activation of stress-response pathways [17,43]. Persistent oxidative damage remains despite adaptive Nrf2 signaling attempts, as substrate availability becomes rate-limiting.

In bulimia nervosa, cyclical restriction-binge-purge patterns create repeated oscillations of Nrf2 activation (during fasting phases) and suppression (during binge episodes involving high-fat, high-calorie intake), potentially leading to maladaptive redox responses and impaired stress resilience [33,48]. In binge eating disorder, particularly when characterized by chronic overconsumption of energy-dense foods without compensatory restriction periods, sustained high-caloric intake and lipid overload suppress Nrf2 activity through multiple mechanisms, including endoplasmic reticulum stress and chronic low-grade inflammation, exacerbating metabolic dysfunction and oxidative stress [48,53].

Additionally, micronutrient deficiencies, common across all restrictive eating disorder phenotypes, may further compromise Nrf2-dependent antioxidant defenses, as many antioxidant enzymes require cofactors such as selenium, zinc, copper, manganese and B vitamins for optimal catalytic function [43]. This distinction is crucial when translating “longevity”- oriented dietary strategies into clinical practice for patients with fibromyalgia and comorbid or latent eating disorders.

## 4. Rehabilitation: The Cornerstone of Integrated Treatment

**Figure 3:**
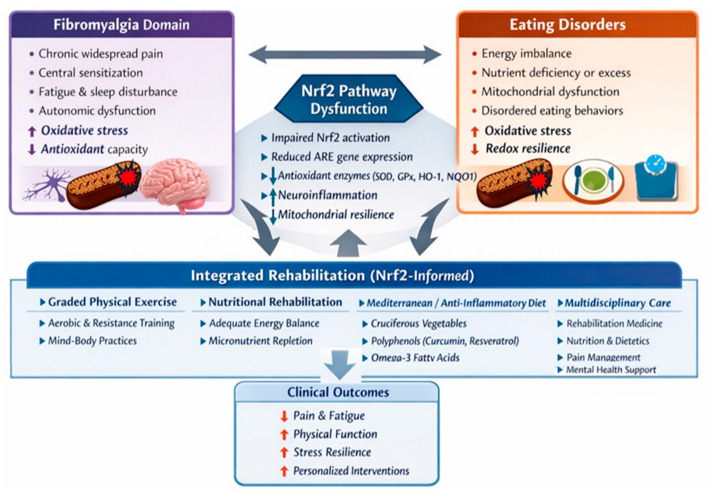
Nrf2-informed rehabilitation framework in fibromyalgia and eating disorders.

Movement fear-avoidance, culminating in a reduction in locomotor activity, frustration over functional limitation, biomechanical/structural changes and lifestyle issues related to diet and sedentary behavior are key components of a progressive loss of functioning in fibromyalgia-associated eating disorders [30,31]. Rehabilitation, therefore, represents a cornerstone of treatment for both eating disorders and fibromyalgia, with emerging evidence suggesting that its therapeutic benefits may be partially mediated through activation of the Nrf2 pathway (Figure 3) [18,19,54,55].

### 4.1. Mechanisms of Exercise and Nutritional Rehabilitation

In eating disorders, nutritional rehabilitation combined with cognitive-behavioral therapy and graduated exercise constitutes the multimodal treatment approach, with evidence demonstrating that it achieves safe weight restoration while reducing complications [56,57]. For fibromyalgia, physical therapy and exercise rehabilitation are strongly recommended as first-line non-pharmacological interventions, with meta-analyses consistently showing that aerobic exercise, resistance training and mind–body practices improve pain, fatigue, physical function and quality of life [58,59].

Mechanistically, both nutritional rehabilitation and physical exercise appear to converge on the Nrf2-Keap1 pathway as a critical mediator of therapeutic benefit [54,60]. Moderate-intensity exercise induces transient increases in reactive oxygen species that activate Nrf2, leading to upregulation of antioxidant defense genes, including superoxide dismutase, catalase, glutathione peroxidase and heme oxygenase-1, thereby enhancing cellular resistance to oxidative stress [54,55,60]. This exercise-induced hormetic stress response, whereby repeated bouts of low-to-moderate oxidative stress promote adaptive antioxidant responses, has been demonstrated across multiple tissues, including skeletal muscle, heart and brain [55,60].

It is worth noting that, while acute exercise activates Nrf2, excessive endurance exercise or exercise to exhaustion can paradoxically suppress Nrf2 activity and increase oxidative damage, emphasizing the importance of appropriately dosed rehabilitation protocols [55,60]. In the context of eating disorders, nutritional rehabilitation that corrects severe energy deficits and micronutrient deficiencies, particularly of selenium, zinc and B vitamins that serve as cofactors for antioxidant enzymes, may restore compromised Nrf2 function, though direct evidence remains limited [43,57].

For fibromyalgia patients, who exhibit impaired Nrf2 activity and elevated oxidative stress markers, graduated exercise rehabilitation represents a non-pharmacological strategy to enhance endogenous antioxidant capacity through Nrf2 activation, potentially addressing a fundamental pathophysiological mechanism underlying chronic pain and fatigue [15,16,18,55,59]. Furthermore, dietary components emphasized in nutritional counseling for both conditions, including polyphenol-rich fruits and vegetables, omega-3 fatty acids and cruciferous vegetables containing sulforaphane, are established Nrf2 activators that may provide synergistic benefits when combined with exercise rehabilitation [18,19,61,62,63].

Thus, multidisciplinary rehabilitation programs incorporating physical therapy, exercise prescription and nutritional optimization offer a promising, mechanism-based approach to address the shared oxidative stress pathophysiology underlying both eating disorders and fibromyalgia, with Nrf2 activation serving as a key molecular target linking these therapeutic modalities to clinical improvement [16,18,19,59].

### 4.2. Nutritional Assessment and Integration

In contemporary pain and rehabilitation medicine, there is growing recognition that nutrition and lifestyle factors are often overlooked components of multimodal programs for fibromyalgia and chronic widespread pain [11,64]. Qualitative and mixed-methods studies in interdisciplinary pain rehabilitation programs indicate that patients commonly report suboptimal dietary habits, emotional or night eating, irregular meal timing and difficulties implementing sustainable lifestyle changes, while expressing a strong desire for more structured and personalized nutritional guidance [29,64]. Integrating systematic nutritional assessment, dietitian-led counseling and eating behavior interventions into fibromyalgia rehabilitation could therefore address important determinants of oxidative stress, metabolic health and treatment adherence that extend beyond traditional physiotherapy and psychological interventions [11,59,64].

## 5. Dietary Nrf2 Activators and Mediterranean Dietary Patterns

Given the critical role of Nrf2 in regulating cellular antioxidant defenses and its potential dysfunction in both fibromyalgia and eating disorders, dietary interventions targeting Nrf2 activation represent a promising, accessible therapeutic approach [18,19]. A growing body of in vitro and clinical evidence demonstrates that numerous phytochemicals and bioactive food compounds can activate the Nrf2-Keap1 pathway, offering a natural mechanism to enhance antioxidant capacity, reduce oxidative stress and potentially ameliorate symptoms in both conditions [18,19,61,62,63,65,66,67]. These dietary Nrf2 activators function primarily through electrophilic modification of critical cysteine residues on Keap1, disrupting its interaction with Nrf2 and allowing nuclear translocation and transcriptional activation of ARE-containing genes [13,14,46,47,61,62].

### 5.1. Cruciferous Vegetables

Cruciferous vegetables, including broccoli, brussels sprouts, cauliflower, kale and cabbage, represent one of the most potent dietary sources of Nrf2 activators, primarily through their high content of glucosinolates that are enzymatically converted to isothiocyanates upon plant tissue disruption [65,67]. Sulforaphane, derived from glucoraphanin in broccoli and particularly abundant in broccoli sprouts, is the most extensively studied dietary Nrf2 activator, demonstrating robust induction of phase II detoxification enzymes and antioxidant proteins in both preclinical and clinical studies [65,67]. Consumption of sulforaphane from broccoli sprout extract has been shown to significantly upregulate NQO1, GST and HO-1 expression in human subjects, with effects persisting for several days after ingestion. The bioavailability and biological activity of sulforaphane can be enhanced through consumption of raw or lightly steamed cruciferous vegetables, as excessive heat denatures myrosinase, the enzyme responsible for converting glucoraphanin to sulforaphane [67].

### 5.2. Polyphenol-Rich Foods

Polyphenol-rich foods represent another major category of dietary Nrf2 activators with substantial evidence for in vitro and clinical antioxidant and anti-inflammatory effects [63,65,66,67,68]. Curcumin, the yellow pigment in turmeric (*Curcuma longa*), is a potent Nrf2 activator that induces HO-1 and other antioxidant enzymes through modification of Keap1 cysteine residues. In vitro studies and clinical investigations in various populations have demonstrated that curcumin supplementation (500–2000 mg/day) can reduce markers of oxidative stress and inflammation in chronic pain populations, though direct evidence in fibromyalgia-specific cohorts remains limited [61,64,69]. Bioavailability limitations have prompted the development of formulations with enhanced absorption, such as curcumin combined with piperine (black pepper extract) [61,64,69].

Resveratrol, found in grapes, red wine, berries and peanuts, activates Nrf2 through multiple mechanisms, including direct Keap1 modification and upstream kinase activation, demonstrating antioxidant, anti-inflammatory and metabolic benefits in preclinical and clinical research [63,70]. Consumption of anthocyanin-rich berries—including blueberries, blackberries, strawberries and cherries—provides multiple polyphenolic Nrf2 activators that have been shown to reduce oxidative stress markers and improve cognitive function in human trials [68,71].

Green tea, particularly rich in the catechin epigallocatechin-3-gallate (EGCG), represents a widely consumed beverage with documented Nrf2-activating properties [72]. EGCG induces Nrf2 nuclear translocation and ARE-dependent gene expression, contributing to the antioxidant and metabolic benefits associated with regular green tea consumption [72]. Clinical studies demonstrate that consumption of 400–800 mg of green tea catechins daily (approximately 3–5 cups of brewed green tea) can enhance antioxidant capacity and reduce oxidative stress markers in human subjects.

Other notable dietary Nrf2 activators include omega-3 fatty acids (particularly EPA and DHA from fatty fish), which modulate Nrf2 activity through both direct and indirect mechanisms [73], allium vegetables (garlic, onions) containing organosulfur compounds, and various herbs and spices, including rosemary, oregano, cinnamon and ginger that contain phenolic compounds with Nrf2-activating properties.

### 5.3. Mediterranean Diet: Evidence-Based Dietary Pattern for Chronic Pain

Higher diet quality, increased intake of fruits, vegetables and whole grains, and reduced consumption of ultra-processed foods are consistently associated with improvements in pain, fatigue and quality of life across chronic pain populations, as demonstrated in systematic reviews of dietary interventions [62,74,75]. Mediterranean-type diets, emphasizing vegetables (including cruciferous varieties), fruits, legumes, whole grains, nuts, seeds, extra-virgin olive oil and fish, provide a practical, evidence-supported framework for nutritional rehabilitation in fibromyalgia and related chronic pain conditions [62,74,75].

The Mediterranean dietary pattern naturally provides a dense matrix of Nrf2-activating phytochemicals, fiber for gut microbiota support and essential micronutrients, while being flexible enough to accommodate individual preferences and tolerances [62,74,75]. Evidence from chronic pain populations and cardiovascular research demonstrates that this dietary approach improves inflammatory markers, oxidative stress biomarkers and patient-reported outcomes (Table 1) [51,64,75,76]. Extra virgin olive oil, a cornerstone of the Mediterranean diet, contains hydroxytyrosol and oleuropein, potent Nrf2 activators that induce HO-1 and NQO1 expression.

Observational studies demonstrate that FM patients adhering to Mediterranean diet patterns report lower pain intensity, reduced fatigue and improved quality of life. A pilot interventional study showed that 12 weeks of Mediterranean diet adherence reduced oxidative stress markers and improved functional capacity in FM patients. The dietary pattern has demonstrated feasibility and acceptability in clinical settings and aligns with established principles of anti-inflammatory nutrition applicable to both fibromyalgia and metabolic rehabilitation contexts [11,76].

### 5.4. Clinical Cautions. Emerging Evidence and the Need for Personalized, Supervised Integration

Although the dietary and nutraceutical strategies discussed in this section are grounded in a coherent mechanistic rationale and supported by preclinical and observational data, it is essential to emphasize that clinical evidence for Nrf2-targeted supplementation specifically in populations with fibromyalgia and eating disorders remains limited and largely indirect. Most intervention data derive from studies in other chronic disease contexts, including metabolic syndrome, cardiovascular disease and cancer prevention, and their direct applicability to comorbid FM–ED populations cannot be assumed without dedicated clinical trials. Furthermore, high-dose supplementation with agents such as curcumin, resveratrol or sulforaphane extracts may carry risks of drug interactions, gastrointestinal side effects, and, in the case of anticoagulant co-administration, increased bleeding risk. In patients with active eating disorders, supplement use may inadvertently reinforce rigid or disordered relationships with food unless carefully framed within a broader therapeutic program. For these reasons, integration of dietary Nrf2 activators and nutraceuticals should always be personalized, medically supervised and embedded within a multidisciplinary care framework. Clinicians are encouraged to apply shared decision-making, consider individual nutritional status and comorbidities, and prioritize whole-food dietary sources over isolated supplements wherever feasible.

### 5.5. Longevity-Oriented Diets: A Double-Edged Sword

Caloric restriction, time-restricted eating, intermittent fasting and ketogenic diets are widely discussed as strategies to promote healthy aging and metabolic health [52,77]. Experimental and translational work suggests that moderate caloric restriction and intermittent fasting can activate Nrf2 and improve mitochondrial function in metabolically healthy populations [52,77]. In obesity and metabolic disease without eating pathology, ketogenic and Mediterranean-ketogenic interventions have shown beneficial effects on weight, glycemic control and inflammatory markers in some populations.

However, when applied in an unselected fashion to FM populations where disturbed eating behaviors and ED are prevalent, these strategies carry substantial clinical risk [5,32,33]. In anorexia nervosa and other restrictive ED, severe energy deficit and micronutrient deficiency are associated with profound oxidative stress and serious medical complications, including cardiac arrhythmias, bone loss and organ dysfunction, imposing further restriction in this context; risks exacerbating both psychiatric symptoms and physiological damage [5,17,33,43]. In bulimia nervosa and binge eating disorder, cycles of restriction and bingeing may destabilize metabolic and redox homeostasis and reinforce loss of control over eating behaviors [33,48].

The key clinical message is that longevity-oriented diets should be considered only in fibromyalgia patients without current or past eating disorders, with careful screening and ongoing monitoring [32]. In individuals with active or a history of ED, the primary nutritional goal must remain restoration or stabilization of adequate energy balance, micronutrient sufficiency and normalization of eating rhythms through regular, balanced meal patterns [5,56,57].

## 6. Clinical Implementation and Screening

In busy fibromyalgia and pain clinics, a single, well-validated self-report screener is more practical than multiple assessment instruments [32,59]. The Eating Attitudes Test (EAT-26) is widely used to detect eating disorder risk and has been adapted to various clinical settings. In clinical practice, EAT-26 can be administered at baseline to identify FM patients at higher risk of eating disorder pathology or disturbed eating patterns [29,32]. A total EAT-26 score ≥20, or high scores on dieting and bulimia-related subscales, should prompt clinical discussion, further detailed assessment and, when appropriate, referral to eating disorder specialists for comprehensive diagnostic evaluation [32].

This pragmatic screening step supports safer nutritional prescriptions, allows appropriate stratification in clinical management and research, and facilitates early intervention [32]. Eating disorder screening should precede the prescription of energy-restricted or ketogenic dietary interventions and be repeated if substantial dietary modifications are introduced during treatment [5,32].

Several biomarkers are commonly used to detect oxidative stress, including isoprostanes, protein carbonyls, peroxides, and antibodies against oxidized low-density lipoprotein (oxLDL). These markers reflect oxidative damage to lipids and proteins and can be measured in biological samples such as blood, urine, or tissues. Isoprostanes are produced by the free radical-induced peroxidation of arachidonic acid in cell membranes and are considered reliable indicators of lipid peroxidation, often used in studies of cardiovascular and metabolic diseases [78]. Protein carbonyls are formed when reactive oxygen species oxidize proteins, creating stable carbonyl groups that serve as markers of oxidative protein damage, commonly assessed in aging and chronic disease research [79]. Peroxides, particularly lipid hydroperoxides, are generated during the early stages of lipid peroxidation and indicate ongoing oxidative processes in cells [80]. Also, antibodies against oxidized LDL (oxLDL) reflect the immune response to oxidatively modified LDL particles and are often studied in relation to atherosclerosis and cardiovascular risk [81]. Together, these biomarkers help assess oxidative damage and the extent of oxidative stress in biological systems and could be useful to evaluate whether altered eating patterns contribute to increased oxidative damage in the body.

## 7. Proposed Integrated Rehabilitation Framework

A Nrf2-informed, eating disorder-aware rehabilitation framework for fibromyalgia patients requires systematic assessment, risk stratification and tailored interventions. Assessment and risk stratification should comprehensively characterize fibromyalgia severity using validated scales (Fibromyalgia Impact Questionnaire, Revised 2016), assess comorbidities, body mass index and metabolic parameters [11]; screen systematically for eating disorder pathology using EAT-26 with follow-up clinical interview when indicated [32]; and evaluate dietary quality and lifestyle patterns using validated nutrition assessment tools [11,64].

For core programs for FM without active eating disorder, clinicians should implement a Mediterranean or anti-inflammatory dietary pattern, rich in vegetables (especially cruciferous vegetables), fruits, whole grains, legumes, nuts, fish and extra-virgin olive oil, with minimization of ultra-processed foods [11,61,62,64,75,76]. Exercise protocols should introduce graded aerobic and resistance training at appropriate intensity (50–70% estimated maximum heart rate for aerobic exercise, progressing as tolerated), complemented by mind–body practices (yoga, tai chi), sleep hygiene optimization and structured stress-management techniques [59,60]. Programs should incorporate Nrf2-activating whole foods throughout the dietary pattern and consider, where appropriate and after shared decision-making, nutraceuticals such as curcumin (500–1000 mg daily in bioavailable formulation) or omega-3 supplementation (1–2 g daily combined EPA/DHA from marine sources) as evidence-based adjuncts (Table 2) [18,66,82].

Adaptations for eating disorder or high-risk profiles fundamentally shift the nutritional focus from weight reduction to restoration or stabilization of adequate nutritional status, establishment of regular meal patterns (typically three meals plus planned snacks) and normalization of eating behaviors. Programs must explicitly avoid fasting protocols, very-low-calorie diets and ketogenic dietary approaches [5,32,56,57]. Close coordination with eating disorder specialists, including registered dietitian nutritionists with specialized training in eating disorder treatment, is essential [11,57]. Pharmacotherapy should be adjusted where necessary, avoiding medications with strong appetite-suppressing properties that may trigger or exacerbate eating disorder symptoms [5,33].

Monitoring and outcomes should track primary outcomes including pain intensity (visual analog scale or numerical rating scale), fatigue severity (Multidimensional Fatigue Inventory), functional capacity (6 min walk test, SF-36 physical function subscale), mood (Hospital Anxiety and Depression Scale), sleep quality (Pittsburgh Sleep Quality Index), and eating behaviors (EAT-26, food frequency records) at baseline, mid-treatment (6–8 weeks) and follow-up intervals (3 months, 6 months) [32,59]. In research settings, objective oxidative stress biomarkers (total antioxidant status, malondialdehyde, oxidative stress index) and Nrf2-related markers should be included where feasible to explore mechanistic links between interventions and clinical improvements [15,38,41].

Nutraceutical safety considerations require attention to potential interactions and contraindications. Curcumin may increase bleeding risk in patients receiving anticoagulant or antiplatelet therapy and should be used cautiously before surgical procedures. High-dose omega-3 supplementation (≥2 g/day EPA/DHA) may similarly potentiate bleeding risk in susceptible individuals [74,82]. Both agents should be used with caution in patients with significant hepatobiliary disease, and all supplement use should be regularly reviewed within a shared decision-making process [18,66,82].

## 8. Limitations

Several considerations should be taken into account when interpreting this narrative review. First, the evidence linking fibromyalgia and eating disorders through oxidative stress and changes in the Nrf2 pathway is mainly indirect. Most studies on Nrf2 signaling, redox imbalance, and mitochondrial function have been carried out in fibromyalgia or eating disorder groups separately, rather than in people who have both conditions. For this reason, the suggested role of Nrf2 is presented as a possible and useful framework for future research, rather than as a confirmed explanation.

Second, research on oxidative stress and Nrf2 activity in eating disorders varies widely and is not evenly spread across diagnoses. Most studies focus on anorexia nervosa, while much less information is available for bulimia nervosa, binge eating disorder, and milder forms of disordered eating. This limits how broadly the findings can be applied and points to the need for studies covering a wider range of eating disorder types.

Third, direct measurements of Nrf2 activity in clinical studies are scarce. Many studies instead use indirect markers of oxidative stress and antioxidant defenses, rather than measuring Nrf2 itself or its target genes. As a result, conclusions about Nrf2 function in patients remain tentative.

Fourth, the rehabilitation and dietary approaches discussed here are based on evidence from related areas such as chronic pain, metabolic health, and nutrition. However, only a small number of randomized controlled trials have tested Nrf2-focused interventions specifically in people with fibromyalgia or eating disorders. The possible benefits of Nrf2-activating foods and exercise-related adaptive responses, therefore, need further study using well-designed clinical trials.

Overall, this review aims to offer an integrated view that links biological mechanisms with clinical and rehabilitation research. The Nrf2-based framework is intended to encourage further studies and to support the development of safe and biologically informed multidisciplinary approaches for people with fibromyalgia and eating disorders.

## 9. Conclusions and Future Directions

The convergence of fibromyalgia and eating disorders represents far more than clinical coincidence—it reflects a biologically plausible and mechanistically compelling interconnection at the molecular level through shared oxidative stress pathophysiology and Nrf2 pathway dysfunction [14,15,16,33,43]. It should be acknowledged, however, that most available evidence derives from studies conducted separately in fibromyalgia or eating disorder populations, and direct clinical data in individuals with comorbid presentations remain limited. The Nrf2-centered framework proposed here is therefore best understood as a promising, evidence-informed hypothesis that warrants rigorous prospective testing, rather than a confirmed unifying mechanism.

This narrative review has synthesized emerging evidence demonstrating that Nrf2, as the master regulator of cellular antioxidant defense, serves as a critical nexus linking the seemingly disparate clinical presentations of chronic pain syndromes and disordered eating behaviors [13,14,16,22]. The documented impairment of Nrf2 activity in fibromyalgia, coupled with oxidative stress dysregulation in eating disorders (albeit with distinct phenotypic patterns), suggests that these conditions may represent different manifestations of a common underlying disturbance in redox homeostasis and cellular stress responses [15,16,17,33,43].

The therapeutic implications of this mechanistic framework are substantial and immediately actionable. Multidisciplinary rehabilitation programs that integrate graduated exercise, nutritional restoration and dietary optimization with Nrf2-activating whole foods offer a scientifically rational, mechanism-based approach that simultaneously addresses both conditions [18,19,55,59,60,61,62,63,66]. The hormetic principle underlying exercise-induced Nrf2 activation—whereby appropriately dosed physical stress triggers adaptive antioxidant responses—provides theoretical and empirical justification for carefully titrated rehabilitation protocols [54,55,60,72]. Similarly, the incorporation of cruciferous vegetables, polyphenol-rich foods, omega-3 fatty acids and other dietary Nrf2 activators into nutritional rehabilitation programs may synergistically enhance endogenous antioxidant capacity while supporting metabolic recovery [18,19,61,62,63,65,66,67,82].

However, significant knowledge gaps persist. The current evidence base remains largely correlational and mechanistic, with limited prospective clinical trials directly testing Nrf2-targeted interventions in comorbid fibromyalgia and eating disorder populations. Critical questions remain unanswered: Does Nrf2 dysfunction represent a shared predisposing vulnerability, or does it emerge as a consequence of chronic pain and nutritional dysregulation? Can targeted Nrf2 activation through dietary or pharmacological means improve clinical outcomes in these populations? What are the optimal dosing, duration and combination parameters of exercise and nutritional interventions to maximize therapeutic Nrf2 pathway activation while preventing maladaptive responses?

Study limitations warrant acknowledgment. First, direct evidence testing the FM-ED-Nrf2 hypothesis in prospective clinical trials remains extremely limited; most supporting data are mechanistic or derived from separate disease populations. Second, the heterogeneity of both fibromyalgia and eating disorder presentations limits the generalizability of Nrf2 dysfunction to all patient subgroups. Third, while Nrf2 pathway activation is well-established for various dietary compounds in vitro and in other disease contexts, their specific effects in FM and ED populations require further investigation. Finally, this synthesis proposes a novel mechanistic framework that warrants rigorous testing through targeted clinical trials before widespread clinical implementation.

Future research priorities should include: (1) prospective longitudinal cohort studies examining Nrf2 pathway biomarkers (nuclear Nrf2 protein, target gene expression, functional antioxidant capacity) across the natural history of fibromyalgia and eating disorders [14,15,16]; (2) randomized controlled trials testing structured, manualized rehabilitation protocols combining appropriately dosed exercise and Nrf2-activating dietary interventions in well-defined FM and ED populations [59,64,76]; (3) mechanistic laboratory studies elucidating the bidirectional relationships between Nrf2 activity, central pain processing, metabolic regulation and eating behavior regulation using translational models [13,14,16,22]; and (4) translational investigations exploring whether pharmacological Nrf2 activators may safely complement behavioral and nutritional rehabilitation strategies in comorbid populations [14,16,18,19].

Clinical implications are immediate and actionable. Clinicians caring for patients with fibromyalgia should maintain heightened clinical awareness for comorbid eating disorder pathology, implementing systematic screening protocols [32]. Conversely, eating disorder specialists should routinely assess for chronic pain conditions and consider their potential impact on treatment engagement and outcomes [5,33]. The recognition that these conditions may share fundamental oxidative stress and Nrf2 pathway dysfunction should inform integrated, holistic treatment approaches that address both pain and eating behavior simultaneously within multidisciplinary, patient-centered care models that prioritize safety, shared decision-making and individualized rehabilitation strategies [18,19,59].

In conclusion, the Nrf2 pathway dysfunction represents a promising and biologically plausible molecular target that may help to unify our understanding of fibromyalgia and eating disorders, pending confirmation from prospective clinical studies in comorbid populations. The convergence of exercise physiology, nutritional biochemistry and redox biology provides a compelling mechanistic framework for optimizing clinical outcomes in these challenging and often comorbid conditions. As our understanding of Nrf2-mediated cellular stress responses continues to evolve, the integration of this knowledge into clinical practice holds substantial promise for improving the lives of individuals struggling with both chronic pain and disordered eating.

## Figures and Tables

**Table 1:** Dietary Nrf2 activators: main food sources, active compounds, typical dosing and evidence.

Food/Source	Main Active Compound(s)	Typical Intake/Dosing Used in Studies	Main Nrf2-Related or Antioxidant Evidence (Population)
Broccoli sprouts, cruciferous vegetables	Sulforaphane (from glucoraphanin)	50–100 g/day fresh sprouts or standardized extract providing ~20–50 µmol sulforaphane	↑ NQO1, GST, HO-1 expression; enhanced detoxification in humans
Mature broccoli, kale, cabbage	Sulforaphane, other isothiocyanates	1–2 portions/day (raw or lightly steamed)	↑ Phase II enzymes, reduced oxidative stress biomarkers
Turmeric (*Curcuma longa*)	Curcumin	500–2000 mg/day curcumin in enhanced-bioavailability formulations	↓ oxidative stress and inflammatory markers in chronic conditions
Green tea	EGCG and other catechins	400–800 mg/day catechins (~3–5 cups brewed tea)	↑ antioxidant capacity, ↓ oxidative stress markers in human trials
Berries (blueberries, blackcurrants, etc.)	Anthocyanins, polyphenols	1–2 portions/day (fresh or frozen)	Improved antioxidant status, endothelial function (various populations)
Grapes, red wine (moderate)	Resveratrol, polyphenols	100–250 mL/day red wine or equivalent grape products	Nrf2 activation and endothelial protection, mainly experimental/human surrogate endpoints
Fatty fish (salmon, mackerel, sardines)	EPA, DHA (omega-3 fatty acids)	~1–2 g/day combined EPA/DHA (diet + supplements)	↓ inflammatory markers, improved symptoms in chronic inflammatory conditions
Garlic, onions	Organosulfur compounds, flavonoids	Regular culinary use	Experimental evidence for Nrf2 activation and antioxidant effects

**Table 2:** Nrf2-informed rehabilitation framework in fibromyalgia with and without eating disorder (ED) risk.

Domain	FM Without Active/Past ED	FM with Active ED or High ED Risk (EAT-26 ≥20 or Clinical Concern)
Primary nutritional goal	Anti-inflammatory, Mediterranean-type dietary pattern; weight management or metabolic risk reduction when indicated	Stabilization or restoration of adequate energy intake; normalization of eating patterns and meal regularity; weight change is secondary to nutritional rehabilitation and ED recovery
Dietary strategy	Mediterranean or anti-inflammatory diet rich in vegetables (including cruciferous varieties), fruits, whole grains, legumes, nuts, fish, and extra-virgin olive oil; minimize ultra-processed foods	Regular structured meals (three main meals plus planned snacks); ensure adequate total energy intake and micronutrient sufficiency; explicitly avoid restrictive protocols including fasting, very-low-calorie diets, ketogenic diets, and intermittent fasting regimens
Nrf2-targeting foods	Actively emphasized: cruciferous vegetables (broccoli, Brussels sprouts, kale), curcumin via turmeric or bioavailable supplements, green tea, omega-3 fatty acids, anthocyanin-rich berries	Nrf2-activating foods integrated only insofar as compatible with safe ED treatment protocols; avoid creating additional dietary restriction or pressure; foods introduced gradually within normalized eating patterns
Nutraceuticals	Consider curcumin (500–1000 mg/day in enhanced-bioavailability formulations) and omega-3 supplementation (1–2 g/day combined EPA/DHA from marine sources), following shared decision-making discussion	Supplements prescribed cautiously and only with explicit written consent from ED treatment team (registered dietitian nutritionist, psychiatrist or psychologist); nutritional food sources prioritized; avoid supplement-dependent approach that may reinforce disordered eating patterns
Physical exercise	Graded aerobic exercise at 50–70% estimated maximum heart rate, resistance training 2–3 times weekly, complemented by mind–body practices (yoga, tai chi, mindfulness); progressive intensity as tolerated	Begin with gentle, body-awareness-promoting movement; avoid exercise framed as caloric compensation or weight management tool; coordinate closely with ED team to ensure exercise does not become compulsive or driven by weight/shape concerns; emphasize rehabilitation and functional recovery
Primary treatment goal	Pain reduction, fatigue improvement, functional capacity enhancement, reduction in cardiometabolic risk factors	ED stabilization and recovery; safe nutritional rehabilitation; psychological and psychiatric treatment integration; FM symptom management subordinate to and coordinated with ED treatment plan
Contraindicated/high-risk strategies	Aggressive caloric restriction without careful assessment for past or latent ED history and systematic EAT-26 screening	Intermittent fasting, ketogenic diets, very-low-calorie approaches; medications with strong appetite-suppressing properties; any dietary intervention implemented without ED specialist involvement and explicit clearance; compulsive exercise protocols

## Data Availability

No new data were created or analyzed in this study. Data sharing is not applicable to this article.

## Abbreviations

The following abbreviations are used in this manuscript:

ARE	Antioxidant response elements
CAT	Catalase
DHA	Docosahexaenoic acid
ED	Eating disorders
EGCG	Epigallocatechin gallate
EPA	Eicosapentaenoic acid
FM	Fibromyalgia
GPx	Glutathione peroxidase
GSTs	Glutathione S-transferases
HO-1	Heme oxygenase-1
HPA	Hypothalamic–pituitary–adrenal
Keap1	Kelch-like ECH-associated protein 1
NQO1	NAD(P)H:quinone oxidoreductase 1
Nrf2	Nuclear factor erythroid 2-related factor 2
ROS	Reactive oxygen species
SOD	Superoxide dismutase
